# Effect of Several Pretreatments on the Lactic Acid Production from Exhausted Sugar Beet Pulp

**DOI:** 10.3390/foods10102414

**Published:** 2021-10-12

**Authors:** Cristina Marzo, Ana Belén Díaz, Ildefonso Caro, Ana Blandino

**Affiliations:** Department of Chemical Engineering and Food Technology, Faculty of Sciences, IVAGRO, University of Cádiz, Campus Universitario de Puerto Real, 11510 Puerto Real, Spain; anabelen.diaz@uca.es (A.B.D.); ildefonso.caro@uca.es (I.C.); ana.blandino@uca.es (A.B.)

**Keywords:** sugar beet pulp, biological pretreatment, alkaline hydrogen peroxide pretreatment, thermochemical pretreatment, enzymatic hydrolysis, lactic acid fermentation

## Abstract

Exhausted sugar beet pulp (ESBP), a by-product of the sugar industry, has been used as a substrate to produce lactic acid (LA). Due to the fact that ESBP contains a high percentage of pectin and hemicellulose, different pretreatments were studied to solubilize them and to facilitate the access to cellulose in the subsequent enzymatic hydrolysis. Several pretreatments were studied, specifically biological, oxidant with alkaline hydrogen peroxide (AHP), and thermochemical with acid (0.25, 0.5, or 1% *w*/*v* of H_2_SO_4_). Pretreated ESBP was enzymatically hydrolysed and fermented with the strain *Lactiplantibacillus plantarum* for LA production. The hydrolysis was carried out with the commercial enzymes Celluclast^®^, pectinase, and xylanase, for 48 h. After that, the hydrolysate was supplemented with yeast extract and calcium carbonate before the bacteria inoculation. Results showed that all the pretreatments caused a modification of the fibre composition of ESBP. In most cases, the cellulose content increased, rising from 25% to 68% when ESBP was pretreated thermochemically at 1% *w*/*v* H_2_SO_4_. The production of LA was enhanced when ESBP was pretreated thermochemically. However, it was reduced when biological and AHP pretreatments were applied. In conclusion, thermochemical pretreatment with 1% *w*/*v* H_2_SO_4_ had a positive impact on the production of LA, increasing its concentration from 27 g/L to 50 g/L.

## 1. Introduction

Lactic acid (LA) is considered one of the top ten green molecules and has recently gained interest because it can be used as the precursor for several other molecules like acrylic acid, which is used for the synthesis of polyacrylates used to produce plastics, paints, fibres, etc., or 2,3-pentanedione, which is used as an aroma or flavouring component in beverages [1]. It is a versatile organic acid that is widely used in the food industry as a preservative (acidifier) and flavour-enhancing agent [2,3,4]. Likewise, LA is extensively employed in cosmetics formulations and the pharmaceutical industry [5]. In addition to these uses, LA has gained interest as a precursor of poly-lactic acid (PLA), which is a bio-degradable and bio-based bioplastic [6].

Lactic acid is usually produced through biotechnological processes by the action of lactic acid bacteria [7]. The main drawback of this process at an industrial scale is the great influence that the raw material has on its economy, due to the use of simple sugars that allow the production of a pure product and reduce purification costs [8]. Recently, lignocellulosic biomasses have been used as a low-cost alternative. Lactic acid fermentation from these materials involves a previous hydrolysis process where the sugars are released from the cell wall of the biomass [9].

Exhausted sugar beet pulp (ESBP), which is one of the main by-products of the sugar industry, is an interesting raw material for LA production. ESBP is the solid residue obtained after the sugar extraction from sugar beet. Nowadays, the pulp is used for animal feed. Thus, it is pressed, dried, and conformed in the form of pellets. Instead, the ESBP can be valorised and used to produce value-added products because it is composed mainly of cellulose, hemicellulose, and pectin. Currently, ESBP has been used as raw material to produce biogas [10], hydrogen [11], ethanol [12], succinic acid [13], lactic acid [14], or enzymes [15]. 

The polysaccharides contained in ESBP are broken down into a mixture of hexoses and pentoses during the enzymatic hydrolysis. The matter is the small number of microorganisms that are able efficiently and simultaneously to convert hexoses and pentoses into lactic acid [16]. To overcome this challenge, pretreatment can be performed before the hydrolysis to obtain a solid rich in cellulose and solubilizing hemicellulose and pectin. Thus, the lactic acid yield produced from ESBP can be improved.

Pretreatment is a stage used before the enzymatic hydrolysis and the main goal is to improve the digestibility of the lignocellulosic biomass by changing its structure and making cellulose more accessible to enzymes. Pretreatments can be classified as physical, chemical, physicochemical and biological. Physical pretreatments reduce the particle size of the biomass and/or the crystallinity of the cellulose in the solid. [17,18]. Chemical pretreatments are performed by the action of alkalis or acids, providing different effects on the pretreated solids. Alkaline pretreatment is focused on the solubilisation of lignin and some hemicelluloses and frequently used reagents like sodium, potassium, calcium, and ammonium hydroxide [19]. On the contrary, acid pretreatment is more effective in solubilising hemicellulose [17] and generally uses inorganic acids, e.g., sulphuric, nitric, or hydrochloric acid. Physicochemical pretreatments combine physical and chemical pretreatments. Biological pretreatments involve using microorganisms such as fungi to degrade lignin and hemicellulose, and in some cases cellulose [17].

Dilute acid pretreatment is the most common pretreatment applied to ESBP. Donkoh et al. conducted this pretreatment on ESBP and reached a hydrolysis yield of 82% [20]. Also, El-gendy et al. optimized the dilute acid pretreatment of ESBP, establishing as best conditions 120 °C with 0.1 N HCl and 14% *w*/*w* of solid loading for 6 min [21]. The authors observed high solubilization of pectin and hemicellulose fractions after this pretreatment, obtaining a yield of 0.14 g of ethanol per gram of ESBP by the alcoholic fermentation of the saccharified pretreated ESBP [21]. Zheng et al. also studied the effect of dilute acid pretreatment on ESBP hydrolysis, obtaining the optimum conditions at 120 °C, 0.66% sulphuric acid, and 6% of solid loading [22]. They performed enzymatic hydrolysis and alcoholic fermentation after the pretreatment, obtaining a hydrolysis yield of 92% and an increase in the ethanol production from 0.16 g to 0.4 g ethanol/g ESBP after the pretreatment [22].

Some papers can be found in the literature as regards the use of acid pretreatment on ESBP. However, most of these studies are focused on the hydrolysis of the pretreated biomass and to release sugars for ethanol production. The novelty of the present research is that it evaluates the influence of different pretreatments of ESBP for lactic acid production. Thus, the present study aimed to improve the lactic acid yield by comparing the effect of different pretreatments on ESBP: biologic, alkaline hydrogen peroxide (AHP), and thermochemical acid. The effect of these pretreatments was evaluated by analysing the fibre composition before and after the pretreatment. Moreover, the effect on the yield of the lactic acid produced from the hydrolysates of the pretreated solids was also studied. 

## 2. Materials and Methods

### 2.1. Raw Material

Exhausted sugar beet pulp (ESBP) was used as raw material to produce lactic acid. ESBP was provided by the British company AB-Sugar located in Jerez de la Frontera (Cadiz, Spain) in the form of pellets. The dried pellets showed a diameter of 6 mm and variable length (10–40 mm). They were composed of 15% molasses and 85% exhausted pulp, being the total solids contents in the range of 80–90%. Samples were collected and stored at –20 °C until use. 

### 2.2. Pretreatment of ESBP

#### 2.2.1. Biological Pretreatment

Biological pretreatment consisted of fungal solid-state fermentation (SSF) of ESBP by *Aspergillus awamori* 2B.361 U2/1, a sequential mutant of *Aspergillus niger* NRRL 3312. Before SSF, spores of *A. awamori* were grown on disposable Petri dishes containing a synthetic medium composed of (g/L): 6 xylan, 5 avicel, 1 pectin, 1 peptone, 0.5 yeast extract, and 15 agar. Petri dishes were incubated at 30 °C for 5 days. After that, spores were suspended in 0.9% *v*/*v* NaCl solution by gentle shaking. The spore concentration was measured with a Neubauer counting chamber. Spores not used were stored in glycerol (50% *v*/*v*) at –25 °C until use. 

SSF was performed in Petri dishes. Hence, 5 g of sterile and dried ESBP was soaked with an appropriate volume of a nutrient solution to adjust the initial moisture content to 70% *w*/*w* and the required volume of spore suspension to obtain a final inoculum concentration of 1 × 10^7^ spores/g of solid. The nutrient solution was composed by (g/L): 2.4 urea, 9.8 g (NH_4_)_2_SO_4_, 5.0 KH_2_PO_4_, 0.001 FeSO_4_·7H_2_O, 0.0008 ZnSO_4_·7H_2_O, 0.004 MgSO_4_·7H_2_O, and 0.001 CuSO_4_·5H_2_O at pH 5. The Petri dishes were incubated at 30 °C for 8 days. The conditions used for SSF were based on our previous research [15].

#### 2.2.2. Alkaline Hydrogen Peroxide Pretreatment

Alkaline hydrogen peroxide (AHP) pretreatment of ESBP was carried out by soaking the solid at a solid/liquid ratio of 1:20 (*w*/*v*) with a solution of 1% *w*/*v* hydrogen peroxide at pH 11.5 (adjusted with NaOH). The mixture was placed in an Erlenmeyer flask (1 L), covered with aluminium foil to avoid the photodegradation of H_2_O_2_, and incubated at 30 °C for 24 h. After that, the slurry was filtered through a Whatman No.1 filter paper and the solid fraction was washed with tap water until a neutral pH was reached to eliminate undesired chemicals. Lastly, the solid was dried in a forced convection oven at 40 °C for 24 h and stored at room temperature until needed for subsequent experiments of enzymatic hydrolysis. The conditions used for AHP pretreatment were based on our previous research [23,24,25].

#### 2.2.3. Thermochemical Pretreatment

Sulphuric acid pretreatment of ESBP was performed by soaking the solid at a solid/liquid ratio of 1:20 (*w*/*v*) with a solution of sulphuric acid at 0.25, 0.5, or 1% *w*/*v*. The mixture was autoclaved at 120 °C for 20 min and, after that, the slurry was filtered through Whatman No.1 filter paper. The solid fraction was washed with tap water until neutral pH. Finally, the solid was dried in a forced convection oven at 40 °C for 24 h and stored at room temperature until needed. Moreover, the same procedure was performed by using distilled water as a control. 

### 2.3. Enzymatic Hydrolysis of Pretreated Solid 

Enzymatic hydrolysis was performed by mixing 6 g (dry wt.) of pretreated ESBP with 60 mL of phosphate buffer (0.05 M, pH 5) in an Erlenmeyer flask (250 mL) and sterilized in an autoclave (120 °C for 20 min). The following enzyme activities of commercial enzymes cocktails were added to the flask: 3.2 FPU per gram of dry biomass (cellulase from *Trichoderma reesei*, Celluclast^®^, Sigma, Darmstadt, Germany), 46.1 U of xylanase per gram of dry biomass (from *Thermomyces lanuginosus*, Sigma), and 46.5 U of exo-polygalacturonase per gram of dry biomass (from *Aspergillus niger*, Sigma). The flask was incubated at 50 °C and 150 rpm for 48 h. Samples were taken throughout the process and stored at –20 °C until use. Each experiment was carried out in triplicate.

### 2.4. Lactic Acid Fermentation of Pretreated ESBP Hydrolysate

*Lactiplantibacillus plantarum* (CECT 748) was used for lactic acid fermentation of ESBP hydrolysates. The strain was maintained as frozen stocks (–70 °C) in Man-Rogosa-Sharpe (MRS) medium supplemented with 15% *w*/*v* glycerol. MRS medium was composed of (g/L): 15 glucose, 10 peptone, 10 meat extract, 5 yeast extract, 5 sodium acetate, 2 ammonium citrate, 0.2 MgSO_4_·7H_2_O, 0.05 MnSO_4_·7H_2_O, and 2 K_2_HPO_4_. 

For inoculum preparation, 6 mL of MRS medium were inoculated with 0.1 mL of the bacterial frozen stocks and incubated in a 10 mL screw-capped test tube (anaerobic conditions) at 30 °C for 24 h. Afterwards, the cultures were propagated two more times in 6 mL of MRS medium with 0.2 mL of the previous culture at the same conditions. In all steps, the concentration of inoculum was 10^7^ cells/mL.

Lactic acid fermentation was performed in ESBP hydrolysates. For this purpose, 60 mL of ESBP hydrolysate were added to a 250 mL Erlenmeyer flask. According to previous studies about the lactic acid fermentation of ESBP hydrolysates [26], the medium was supplemented with 5 g/L of yeast extract, as a source of nitrogen, and 18 g/L of CaCO_3_ for pH control. The pH of the suspension was adjusted to 6.5. After adding 0.6 mL of *L. plantarum* inoculum to the flask, it was incubated at 30 °C and 150 rpm for 7 days. Samples were collected throughout the fermentation and stored at –25 °C for analysis. Fermentations were carried out in triplicate.

### 2.5. Analytical Techniques

#### 2.5.1. Determination of Fibre Composition

Detergent fibre analysis of the solids was carried out following the standard methods. The methodology described in EN ISO 13906:2008 was employed to determine acid detergent fibre (ADF) and acid detergent lignin (ADL), while the one described in AOAC 2002:04/ISO 16472:2006 was used to determine amylase-treated neutral detergent fibre (aNDF). Both methods were carried out in Fibertec™ 8000 (FOSS IBERIA, Barcelona, Spain) y FT 121 Fibertec (FOSS IBERIA, Barcelona, Spain). The former was used for ADL determination and to analyse fats, while the second one was used for the analysis of aNDF and ADF.

Solid samples were milled until the obtention of a particle size smaller than 1 mm. Then, they were dried in an oven (2 h, 105 °C) to remove the humidity. Finally, they were kept at room temperature in a desiccator for 30 min.

This methodology allows the quantification of the following fractions: removable with acetone (fats, oils, etc.), removable with neutral detergent (proteins, enzymes, pectin, etc.), removable non-calcined (soluble salts), removable calcined (rest of extractable material but not saline), removable with acid detergent (hemicellulose), extractable with concentrated acid (cellulose and soluble lignin), non-removable but calcined (insoluble lignin), non-removable and non-calcined (insoluble salts), and finally a fraction of totally calcined (total salts). Analyses were made in triplicate.

#### 2.5.2. Determination of Moisture Content of ESBP

ESBP moisture content was determined by drying a proper amount of ESBP until constant weight in an oven at 105 °C. The moisture content was calculated as follows: percent moisture content (initial) of ESBP = (weight of ESBP − dry weight) × 100/dry weight.

#### 2.5.3. Determination of Sugar Concentration

Samples collected during enzymatic hydrolysis and fermentations were centrifuged at 10,000 rpm for 10 min at 4 °C. The supernatant was collected to analyse the concentration of reducing sugars and simple sugars. Reducing sugars concentration was measured by a modification of the 3,5-dinitrosalicylic acid method (DNS) adapted to microplates [27,28]. 

Simple sugars were measured by ion chromatography (Metrohm 930 Compact IC Flex, Herisau, Switzerland) with a pulse amperometric detector with a gold electrode as a working electrode. Elution was carried out in isocratic at a 0.35 mL/min flow rate with 300 mM sodium hydroxide (NaOH) and 1 mM sodium acetate (NaOAc). Separation was achieved with two sequential columns: Metrosep Carb 2-150/4.0 and Metrosep Carb 2-250/4.0 (Metrohm, Herisau, Switzerland).

The monosaccharide contents in samples were determined by comparing their retention times against the retention times of the pure standards. Standard curves of the following sugars were used to quantify the sugar concentration (linear range used in g/L): glucose (0.4–2 × 10^−5^), xylose (0.1–5 × 10^−5^), arabinose (0.1–5 × 10^−5^), galactose (0.1–5 × 10^−5^), fructose (0.4–5 × 10^−5^), lactose (0.1–5 × 10^−5^), cellobiose (0.1–5 × 10^−5^), and saccharose (0.1–5 × 10^−5^). Sugars measurements were performed by using the Peripheral Research Services of IVAGRO at the University of Cádiz.

#### 2.5.4. Determination of Organic Acid Concentration 

Samples collected during fermentations were centrifuged at 10,000 rpm for 10 min at 4 °C. The supernatant was collected to analyse organic acids. Organic acids (lactic acid and acetic acid) were measured by ionic chromatography (Metrohm, 930 Compact IC Flex, Herisau, Switzerland) with conductivity detection and a Metrosep Organic Acids-250/7.8 column (Metrohm, Herisau, Switzerland). The separation was carried out using as eluent a solution composed of 0.4 mmol/L sulphuric acid and 0.12 mL/mL acetone, at an isocratic flow rate of 0.4 mL/min.

The organic acids content in samples were determined by comparing their retention times against the retention times of the pure standards. Standard curves of the following organic acids were used to quantify the organic acid concentration (linear range used in g/L): citric acid (0.2–1 × 10^−3^), galacturonic acid (0.2–1 × 10^−3^), lactic acid (0.2–1 × 10^−3^), and acetic acid (0.2–1 × 10^−3^). Organic acids measurements were performed by using the Peripheral Research Services of IVAGRO at the University of Cádiz.

#### 2.5.5. Determination of Cell Growth in Lactic Acid Fermentation

The cell growth during LA fermentations was measured using the colony-forming unit (CFU) counting method. Samples taken during fermentations were serially diluted in NaCl solution (9 g/L) and cultured in MRS-agar plates, which were incubated in static conditions at 30 °C for 48 h.

#### 2.5.6. Statistical Analysis

All experiments and assays were performed in triplicate. Statgraphics 18 was used for data analysis. Data were analysed using one-way analysis of variance (one-way ANOVA) and Fisher’s least significant differences (LSD, *p* < 0.05) was used to determine significant differences among tested conditions.

## 3. Results and Discussion

### 3.1. Pretreatment Effects on the Fibre Composition

**Figure 1:**
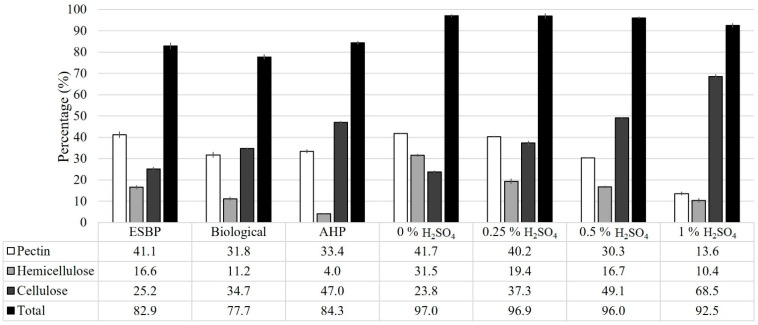
Fibre composition of exhausted sugar beet pulp (ESBP) in dry basis (% *w*/*w*): non-pretreated ESBP (ESBP), biologically pretreated ESBP (biological), alkaline hydrogen peroxide pretreated ESBP (AHP) and thermochemical acid pretreated ESBP with sulphuric acid (H_2_SO_4_) using four concentrations (0, 0.25, 0.5 and 1% *w*/*v*). The “total” shows in the figure is the sum of pectin, hemicellulose and cellulose, not being considered other components like lignin, salts or fats. The sum of all the components is 100% *w*/*w* in all the pretreatments.

The non-pretreated and pretreated ESBP were subjected to a fibre analysis. The results obtained are shown in Figure 1, where the percentages (% *w*/*w*) of pectin, hemicellulose, and cellulose are represented in a bar diagram. These results show how each of the three pretreatments tested affected the fibre composition of ESBP. Firstly, it can be observed that the total percentage of hydrolysable polysaccharides (the sum of cellulose, hemicellulose and pectin) decreases from 82.9% *w*/*w* to 77.7% *w*/*w* when the solid was biologically pretreated. In this case, the pretreated ESBP showed a loss of 22.8% in pectin and 32.8% in hemicellulose and as a consequence, the content in cellulose increases to 34.7% *w*/*w*. During SSF, the fungus secreted hydrolytic enzymes, such as xylanases and pectinases, which mainly hydrolysed the hemicellulose and pectin fractions, and the released sugars were used for fungal growth [15]. For this reason, these fractions are reduced in the biological pretreatment. Similar results were observed by Shi et al. in the biological pretreatment of cotton stalks with *Phanerochaete chrysosporium*. They observed a reduction in hemicellulose of around 60% after 14 days of fermentation, due to the action of the xylanases produced by the fungus [29]. Another study performed by Wan and Li resulted in a loss of 22% of hemicellulose when corn stover was pretreated with *Ceriporiopsis subvermispora* for 18 days [30]. 

Secondly, in the solid pretreated with alkaline hydrogen peroxide, the total percentages of hydrolysable polysaccharides were similar to the non-pretreated ESBP (84.4% *w*/*w* vs. 82.9% *w*/*w*), with the differences being not statistically significant (*p*-value > 0.05). However, it can be observed that the percentage of cellulose increases to 47.0% *w*/*w* while the percentage of hemicellulose decreases to 4.0% *w*/*w* (75.8% of hemicellulose loss). Additionally, the percentage of pectin dropped to 33.4% *w*/*w* (18.9% of pectin loss). Thus, this pretreatment was effective in the solubilisation of the hemicellulose, while the cellulose is hardly affected. In the literature, it is established that AHP pretreatment affects mainly the lignin fraction of the lignocellulosic biomass. However, the low content of lignin in ESBP can lead to the solubilization of the hemicellulose fraction [31]. Park and Kim also observed the solubilization of hemicellulose fraction when various alkaline pretreatments were applied on different lignocellulosic biomass, such as eucalyptus residue, *Larix leptolepis*, *Pinus rigida*, rice straw, and barley straw [32]. They found solubilisation percentages between 5% and 13%, depending on the alkaline pretreatment and the lignocellulosic biomass used. Comparing the results obtained by Park and Kim with the ones of this study, a similar percentage of hemicellulose solubilization was measured (12%).

Regarding the thermochemical pretreatment with sulphuric acid, several concentrations of acid were studied (0, 0.25, 0.5, and 1% *w*/*v* H_2_SO_4_). In this case, it can be seen how the total percentage of hydrolysable polysaccharides increases when the solid was pretreated with distilled water (0% *w*/*v* H_2_SO_4_) compared to the non-pretreated ESBP (97% *w*/*w* vs. 83% *w*/*w*). On the contrary, when 1% *w*/*v* H_2_SO_4_ was added, a slight reduction in hydrolysable polysaccharides was observed (97% *w*/*w* vs. 93% *w*/*w*), while the percentage of cellulose increased by 69% *w*/*w* (vs. 25% *w*/*w* on non-pretreated ESBP). This increase was the consequence of the reduction of pectin and hemicellulose content due to their solubilisation. Consequently, the fraction of pectin decreased when 1% *w*/*v* H_2_SO_4_ was used, from 41% *w*/*w* to 14% *w*/*w* and hemicellulose from 17% *w*/*w* to 10% *w*/*w*. Comparing 0% and 0.25% *w*/*v* H_2_SO_4_ hydrothermal pretreatments, hemicellulose content is reduced significantly (from 32% *w*/*w* to 19% *w*/*w*) but not pectin (from 42% *w*/*w* to 40% *w*/*w*). Such findings were expected because ESBP contains a high amount of amorphous hemicellulose which can be easily hydrolysed by dilute acids at temperatures in the range of 110–140 °C [33,34]. On the other hand, cellulose is more recalcitrant towards dilute acid hydrolysis at such temperatures, being practically unchanged up to 170 °C and only efficiently hydrolysed at temperatures greater than 240 °C [34,35]. Similar results were obtained by Canilha et al. They performed an optimization of the dilute sulphuric acid pretreatment of sugarcane bagasse considering three variables: temperature, acid concentration, and time. Almost complete removal of the hemicellulose fraction, with low cellulose solubilisation, was reached with 2.5% *w*/*v* of H_2_SO_4_ at 150 °C for 30 min [33]. As in our study, Canilha et al. observed the lowest solid solubilization when it was pretreated in absence of acid, confirming that an acid catalyst is necessary to degrade hemicellulose at moderate temperatures [33,36].

### 3.2. Pretreatment Effects on Enzymatic Hydrolysis and Lactic Fermentation

All pretreated solids were hydrolysed and subsequently fermented with *L. plantarum*. Briefly, the enzymatic hydrolysis was performed by suspending 6 g of pretreated ESBP in 60 mL of the appropriated buffer and adding Celluclast^®^ (Sigma, Darmstadt, Germany) supplemented with commercial xylanase and pectinase. The suspension was incubated at 50 °C for 48 h. Afterwards, the hydrolysate obtained was supplemented with yeast extract and CaCO_3_ and the pH was adjusted to 6.5. Finally, it was inoculated with *L. plantarum* and incubated at 30 °C. 

**Figure 2:**
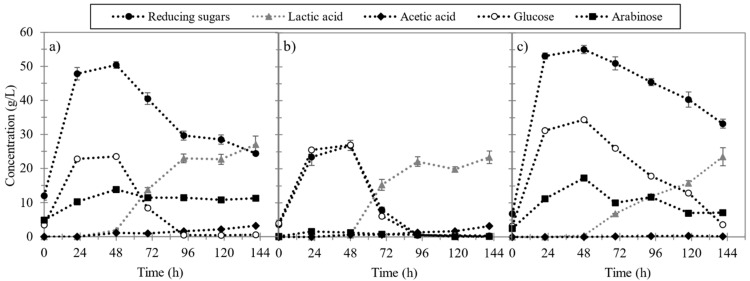
Enzymatic hydrolysis and lactic acid fermentation of non-pretreated ESBP (**a**), biologically pretreated ESBP (**b**) and alkaline hydrogen peroxide pretreated ESBP (**c**): reducing sugars concentration (black circle), lactic acid concentration (grey triangle), acetic acid concentration (black diamond), glucose concentration (white circle) and arabinose concentration (black square).

**Figure 3:**
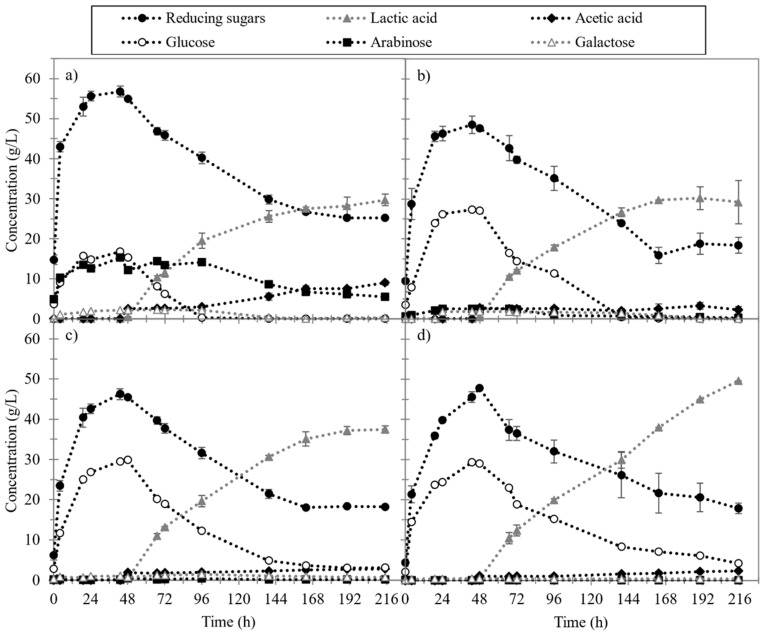
Enzymatic hydrolysis and lactic acid fermentation of thermochemical pretreated ESBP at sulphuric acid concentration of 0% (**a**), 0.25% (**b**), 0.5% (**c**) and 1% *w*/*v* H_2_SO_4_ (**d**): reducing sugars concentration (black circle), lactic acid concentration (grey triangle), acetic acid concentration (black diamond), glucose concentration (white circle), arabinose concentration (black square) and galactose concentration (white triangle).

The results obtained are shown in Figure 2 and Figure 3. For non-pretreated solid (Figure 2), the maximum reducing sugars concentration (50 g/L) was obtained after 48 h of hydrolysis and the maximum lactic acid concentration (27 g/L) after 144 h. After 48 h of hydrolysis, the hydrolysate was composed of glucose (23 g/L) and arabinose (13 g/L). For the biologically pretreated ESBP (Figure 2), 48% less RS concentration was produced (26.7 g/L), while the maximum lactic acid concentration decreased by 15% (23 g/L). These differences could be related to the decrease in the percentage of total hydrolysable polysaccharides of the biologically pretreated ESBP. However, despite this reduction in the RS concentration, most of them were glucose, being consumed in the lactic acid fermentation, as it is the main sugar fermented by *L. plantarum* [26]. Thus, the hydrolysate obtained from the biologically pretreated ESBP is richer in glucose than the one obtained from the non-pretreated solid, due to its higher content in cellulose. In this way, the concentration of lactic acid produced is only reduced by 15%. Although there is a reduction in the percentage of total hydrolysable polysaccharides after the biological pretreatment, a high percentage of hemicellulose and pectin are not solubilized. Similar findings were reported by Shi et al. for the enzymatic hydrolysis of biological pretreated cotton stalks with *Phanerochaete chrysosporium* [29]. They also observed that the concentration of sugars released in the hydrolysis of the pretreated solid was lower than for the non-pretreated solid. Shi et al. stated that the spreading of fungal mycelia and the attachment on cotton stalks of the ligninolytic enzymes produced by the fungus during the microbial pretreatment hinders the binding of cellulolytic enzymes onto cellulose [29]. In the present study, it seems that the fungus *A. awamori* grows using mainly hemicellulose and pectin fractions of ESBP, leaving cellulose untouched. Moreover, it was observed in our previous studies that the fungus produced low cellulase activity during solid-state fermentation [15]. 

Comparing the results obtained for AHP pretreated ESBP with non-pretreated ESBP (Figure 2), maximum RS concentration rose by 10% (55 g/L) while maximum LA concentration fell by 15% (23 g/L). These results are in line with the increase in the percentage of total hydrolysable polysaccharides observed in fibre composition analysis. The slight reduction in the maximum concentration of lactic acid and the slower fermentation rate may indicate the presence of inhibitors, such as 5-hydroxymethylfurfural or furfural, produced from sugar degradation during oxidant pretreatments [37]. However, the percentage of cellulose in AHP pretreated ESBP is higher compared to the non-pretreated and biologically pretreated solids and, therefore, higher glucose concentration (34 g/L) was produced and higher lactic concentrations would be expected. From the shape of the lactic acid production curve, it can be deduced that the fermentation is not yet finished and the concentration of lactic acid tends to continue to rise. So, the maximum LA concentration is likely to be reached at a longer fermentation time. 

Regarding the results obtained with the thermochemical pretreatment (Figure 3), various concentrations of sulphuric acid were tested (0, 0.25, 0.5, and 1% *w*/*v* H_2_SO_4_). Compared to non-pretreated ESBP (50 g/L of RS and 27 g/L of LA), with 0% *w*/*v* H_2_SO_4_, the maximum concentration of reducing sugars and lactic acid increased by 8% (54 g/L) and 7% (29 g/L), respectively (Figure 3a). However, for 0.25 and 0.5% *w*/*v* H_2_SO_4_, the RS concentration decreased by 6% and 10% (47 and 45 g/L) while lactic acid increased by 11% and 37% (30 g/L and 37 g/L), respectively. Finally, for 1% *w*/*v* H_2_SO_4_, RS also decreased by 10% (45 g/L) but lactic acid increased substantially (50 g/L). In summary, as the sulphuric acid concentration increases, the maximum achievable sugar concentration drops and the LA concentration rises. This effect can be explained with the fibre composition, given that the percentage of total hydrolysable polysaccharides is reduced as the concentration of sulphuric acid is increased. Although compared to non-pretreated ESBP the percentage of total hydrolysable polysaccharides is higher for all the sulphuric acid concentrations tested, the maximum RS concentration reached is only higher for 0% *w*/*v* H_2_SO_4_. This effect could be due to the most suitable enzymatic cocktail was not used in all the conditions. The same enzymatic cocktail was added at different sulphuric acid concentrations. However, huge differences were found on the composition of ESBP pretreated with different concentrations of this acid. Regarding the maximum concentration of lactic acid produced, it can be observed that it can be increased at least until 50 g/L for 1% H_2_SO_4,_ and an even higher concentration of LA could be achieved at longer fermentation times. Despite the solid showing a lower content of hydrolysable polysaccharides at 1% H_2_SO_4_, it contains a high percentage of cellulose (69%), which can be hydrolysed to glucose and fermented to lactic acid. It can also be observed that the maximum LA concentration increases, and the maximum acetic acid concentration decreases, with the amount of sulphuric acid added. This is also in agreement with the differences in the fibre composition and consequently in the type of sugar available in the hydrolysate for the subsequent fermentation. So, the concentration of acetic acid decreases at a higher concentration of sulphuric acid because it is generally produced by *L. plantarum* in the fermentation of arabinose [26], a sugar present in hemicellulose, whose content is lower in these conditions. 

The results obtained in the enzymatic hydrolysis of the thermochemically pretreated ESBP were compared with the ones obtained by other authors. Wei et al. also observed that the thermochemical acid pretreatment of eucalyptus chips released a hydrolysate richer in glucose when the concentration of sulphuric acid on the pretreatment was increased [38]. They observed that a higher fraction of hemicellulose was solubilized when the pretreatment was carried out at higher acid concentrations, temperature, or pretreatment time, obtaining a hydrolysate richer in glucose rather than a mixture of glucose and xylose. They also establish that the optimum acid concentration to pretreat eucalyptus chips was 0.75% *w*/*w* because higher concentrations of acids produced a lower concentration of glucose in the hydrolysate, probably due to the cellulose solubilization during the pretreatment [38]. Similar results were obtained by Tang et al. when wheat straw was thermochemically pretreated with dilute sulphuric acid and enzymatically hydrolysed, producing a hydrolysate richer in glucose when the acid concentration used in the pretreatment was increased [39].

In the present work, it was also observed that the hydrolysis rate was lower when the solid was pretreated with high concentrations of sulphuric acid. This can be due to the proportion of the different enzyme activities in the cocktail added to the pretreated solid was not adequate. The solid obtained after the pretreatment with 1% *w*/*v* H_2_SO_4_ shows a high content of cellulose and a reduced content of hemicellulose and pectin. On the contrary, the solid pretreated with 0% *w*/*v* H_2_SO_4_ shows a higher percentage of pectin and hemicellulose than cellulose. Therefore, the solid pretreated with 1% *w*/*v* H_2_SO_4_ will probably need an enzymatic cocktail with a higher cellulase content and lower pectinase and xylanase activity.

Comparing all the pretreatments studied, it seems that the production of lactic acid can be notably improved (as much as 85% compared with non-pretreated ESBP) with the thermochemical acid pretreatment at 1% *w*/*v* H_2_SO_4_. In addition, the application of this pretreatment on ESBP can make the process more viable on an industrial scale. The industrial production of lactic acid from lignocellulosic biomass is usually performed in four stages: pretreatment, enzymatic hydrolysis, fermentation, and product extraction and purification [1]. Conventionally, several steps of purification are needed after fermentation to produce the final lactic acid product, accounting for up to 50% of the production cost [40,41]. Comparing the results obtained in this study with and without the pretreatment stage, a more concentrated and purer product is obtained when ESBP is pretreated with 1% *w*/*v* H_2_SO_4_. This result would decrease the costs of extraction and purification of the lactic acid and, although the application of the pretreatment would increase the production costs, the high concentration of lactic acid obtained could make the process feasible on an industrial scale. Moreover, the raw material cost is reduced due to the use of a by-product of the sugar industry (ESBP). However, future studies would recommend the use of a microorganism that produces a pure optical isomer of L(+) or D(-)-lactic acid, as the strain selected in this study produces a racemic mixture of lactic acid. The pure isomers have greater value than the racemic mixture because they are used for specific industrial applications, e.g., L(+)-lactic acid is used in the synthesis of L(+)-polylactic acid, a biodegradable semi-crystalline and thermosetting polymer [42].

## 4. Conclusions

The pretreatments applied on exhausted sugar beet pulp (ESBP) change its fibre composition, increasing the relative cellulose content. Alkaline hydrogen peroxide pretreatment causes mainly hemicellulose solubilisation while the thermochemical with sulphuric acid and the biological pretreatments solubilise most of hemicellulose and pectin, increasing the cellulose content up to 68.5% *w*/*w* and 34.7% *w*/*w*, respectively. These changes in the fibre composition of the pretreated ESBP produce different results in the lactic acid fermentation of its hydrolysates. Thus, the fermentation of hydrolysates from biologically and AHP pretreated ESBP produces lower lactic acid contents than that from the thermochemically acid pretreated pulp because the glucose content is lower. From the results obtained, it seems that the best pretreatment to be applied on ESBP to obtain a hydrolysate rich in glucose and with a consequently high concentration of lactic acid (of at least 50 g/L) with *Lactiplantibacillus plantarum* fermentation is thermochemical with sulphuric acid at 1% *w*/*v*.

## Data Availability

Not applicable.
